# Investigation of Thermal and Spectroscopic Properties of Tellurite-Based Glasses Doped with Rare-Earth Oxides for Infrared Solid-State Lasers

**DOI:** 10.3390/ma17153717

**Published:** 2024-07-27

**Authors:** Ahlem Boussetta, Aref M. Al-Syadi, Kamel Damak, Ali Erçin Ersundu, Miray Çelikbilek Ersundu, Essam Ramadan, Ali M. Alshehri, Khalid I. Hussein, Ramzi Maalej, El Sayed Yousef

**Affiliations:** 1 Laboratory of Systems Integration and Emerging Energies, National Engineering School of Sfax (ENIS), University of Sfax, Sfax 3018, Tunisia; ahlem.bousseta@ipeis.usf.tn; 2 Department of Physics, Faculty of Science and Arts, Najran University, Najran 11001, Saudi Arabia; arefalsyadi@yahoo.com; 3 Promising Centre for Sensors and Electronic Devices (PCSED), Advanced Materials and Nano-Research Najran University, Najran 11001, Saudi Arabia; 4 LaMaCoP, Faculty of Sciences of Sfax, University of Sfax, Sfax 3018, Tunisia; kamel.damak@ipeis.usf.tn (K.D.); ramzi.maalej@fss.usf.tn (R.M.); 5 Department of Metallurgical and Materials Engineering, Yildiz Technical University, Davutpasa Campus KMA-205, Esenler, Istanbul 34220, Turkey; ersundu@yildiz.edu.tr (A.E.E.); miray@yildiz.edu.tr (M.Ç.E.); 6 Physics Department, Faculty of Science, Al Azhar University, Assuit Assiut 71542, Egypt; esam_ramadan2008@yahoo.com; 7 Department of Physics, Faculty of Science, King Khalid University, Abha P.O. Box 9004, Saudi Arabia; amshehri@kku.edu.sa (A.M.A.); ayousf@kku.edu.sa (E.S.Y.); 8 Department of Radiological Sciences, College of Applied Medical Sciences, King Khalid University, Abha 61421, Saudi Arabia

**Keywords:** co-doped tellurite glasses, rare earth, DSC, thermal properties, refractive index, Judd–Ofelt analysis, gain quality

## Abstract

The thermal and optical properties of 60TeO_2_-20K_2_TeO_3_-10WO_3_-10Nb_2_O_5_ (in mol%) glasses doped with Ho_2_O_3_, Er_2_O_3_, and Tm_2_O_3_ were explored in the present work. The thermal stability, refractive index n, extinction coefficient k, absorption coefficient α, and optical band gap of the glasses were evaluated. The UV–Vis–NIR absorption spectra, the Judd–Ofelt intensity parameter, the spectroscopic quality factor, and the emission and absorption cross-sections were calculated to investigate the effects of Er^3+^ and Tm^3+^, respectively, on the band spectroscopic properties of Ho^3+^ ions. The results showed that the maximum emission cross-section was approximately $8\times 10^{-21}\;{cm}^{2}$, and the values of the full width at half maximum (FWHM), quality factor ($\sigma_{e}\times FWHM)$, and gain coefficient of Ho^3+^: ^5^I_7_→^5^I_8_ were also reported. The value of the $FWHM\times \sigma_{e}$ was $1200\times 10^{-28}\;{cm}^{3}$, which showed greater gain characteristics than earlier study results. For 2 μm mid-infrared solid-state lasers, the glasses that were examined might be a good host material.

## 1. Introduction

Lasers, optical fiber amplifiers, flat-panel displays, optoelectronics, memory devices, solar cells, and light-emitting diodes are just a few of the numerous technical and scientific uses of glasses [1,2]. Oxide glasses often have several desirable characteristics, including low optical properties thresholds, and great transparency. Several researchers have been actively working to change these materials to acquire significant nonlinear coefficients, which are needed for using them in nonlinear optical devices [1,2,3]. It is well known that tellurite-based glasses are transparent in the mid-infrared range, have a high refractive index, and a high density. Not only are these glasses non-toxic, but they also resist moisture and are stable against devitrification. These characteristics make the glasses useful in many fields, including laser windows, nonlinear optical devices (including limiters, optical switches, and modulators), and optical fibers [4]. The addition of rare-earth ions is essential for white-light-emitting diodes, and glass systems based on TeO_2_ are very promising matrices for this purpose [5]. There has been a recent renaissance in the study of earth-doped materials for photonic applications, display monitors, X-ray imaging, scintillators, lasers, up-conversion, and amplifiers for fiber-optic communications [5,6,7]. Rare-earth-doped glasses have important applications in solid laser sources, optical sensors, solar cells, optical telecommunication, white-light-emitting diodes, and optical data storage devices [7,8]. Tellurite glasses with added transition metal oxides should have a higher softening point and more stability, as seen with WO_3_ [9]. The WO_3_ and TeO_2_ components of the glass are network formers. WO_4_ tetrahedral and WO_6_ octahedral structural units and TeO_3_ trigonal pyramid and TeO_4_ trigonal bipyramid units are the two different kinds of dopant sites that are present in the glass because of this [10]. The two different types of dopant sites vary in the intensity of their ligand fields and, as a result, in their distributions in space due to their unique geometries [5]. According to Pandey et al. [6], the third nonlinearity effect is due to the ability of WO_3_ to increase the density of the non-bridging oxygen atoms, which causes the optical band gap to increase in Bi_2_O_3_-WO_3_-TeO_2_ glasses. According to Kim and Yoko [11], the optical band gap, third-order susceptibility, χ^(3)^, real refractive index, static refractive index, and empty d-shell transition metal cations, including Nb^5+^ cations, affect oxide glasses.

The present work aims to study the optical properties of the TeO_2_-K_2_TeO_3_-WO_3_-Nb_2_O_5_ glass system, doped with rare-earth oxides (Ho_2_O_3_, Er_2_O_3_, and Tm_2_O_3_). The optical characteristics are correlated with the structure’s phase transitions and thermal stability, which are determined using differential scanning calorimetry and a double-beam spectrophotometer. The ultraviolet–visible–near-infrared (UV–Vis–NIR) absorption and emission spectra of Ho^3+^-single-doped, Ho^3+^/Er^3+^-co-doped, and Ho^3+^/Tm^3+^-co-doped tellurite glasses (TKWN1, TKWN2, and TKWN3, respectively) were analyzed at room temperature. Based on the Judd–Ofelt theory, the detailed spectroscopic parameters, radiative transition probabilities, radiative lifetimes, and branching ratios of the TKWN1 sample were obtained. Moreover, the absorption and emission cross-sections of Ho^3+^ were calculated via McCumber theory. In addition, the gain coefficient of Ho^3+^: ^5^I_7_→^5^I_8_, the quality factor ($\sigma_{e}^{peak}\times FWHM)$, and the full width at half maximum (FWHM) were also reported. Finally, the possible visible and NIR emissions and their applications for future green laser sources and optical amplifiers were discussed.

## 2. Experimental Section

**Figure 1:**
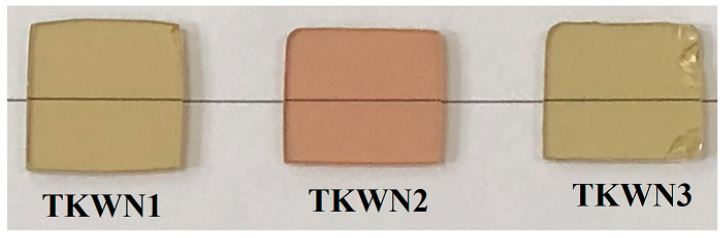
Photographs of as-prepared TKWN glasses.

Using the melt quench technique, tellurite-based glasses with the composition (60TeO_2_-20K_2_TeO_3_-10WO_3_-10Nb_2_O_5_) mol%, doped with rare-earth oxides (Ho_2_O_3_, Er_2_O_3_, and Tm_2_O_3_ in ppm ratio), were prepared. The details of their composition are shown in Table 1. The powder was mixed and heated in a platinum crucible in a furnace at 960 °C for 35 min. Subsequently, the highly viscous melt was cast into a graphite mold. The quenched glass was annealed at 250 °C for 2 h and then slowly cooled to room temperature (RT). The color of the prepared samples depended on the ratio of NiO in the glasses (as clarified in Figure 1). The thermal analysis of the glasses was carried out via differential scanning calorimetry (DSC Shimadzu 50) with a heating rate of 10 °C/min in the range of 20–550 °C. The density of the glass samples was evaluated using the Archimedes method.

(1) $$\rho =\frac{W_{air}\times \rho_{l}}{\left(W_{air}-W_{l}\right)}$$

where W_air_ and W_L_ are the weights of the glass sample in air and toluene, respectively. $\rho_{l}$ is the density of toluene liquid ($\rho_{l}=0.865\;gm\cdot {cm}^{3})$.

The optical absorption and transmission spectra were measured in the wavelength range of 400–2500 nm using aJASCO V-570 spectrophotometer (JASCO INTERNATIONAL CO., LTD. Tokyo, Japan).

## 3. Results and Discussion

### 3.1. Density, Molar Volume, and Oxygen Packing Density

The density (*ρ*), molar volume (*V*_m_), and oxygen packing density (OPD) of the studied glasses are listed in Table 1. The density of the glasses varied between 4.9125 and 4.9641 g/cm^3^. The addition of rare-earth oxides to TeO_2_-based glasses leads to a relatively slight increase in density. This increase is related to the molecular weights of rare-earth oxides, which are much greater than those of other constituents in the studied TeO_2_-based glass, and also to the change in the coordination number of rare-earth ions [12,13]. Therefore, the densities of the glasses co-doped with the two types of rare-earth oxides, Ho_2_O_3_ and Er_2_O_3_ (TKWN2 sample) and Ho_2_O_3_ and Tm_2_O_3_ (TKWN3 sample), were relatively higher than the glass doped with Ho_2_O_3_ only (TKWN1 sample). Furthermore, the molecular weights of Er_2_O_3_ and Tm_2_O_3_ (382.5 and 385.866 g/mol, respectively) are higher than that of Ho_2_O_3_ (377.858 g/mol), which led to an increase in the density of the TKWN2 and TKWN3 glasses compared to the TKWN1 glass. Given that the density is inversely proportional to the molar volume and proportional to the average molecular weight, it is reasonable to assume that the two quantities will behave in opposition to one another in most amorphous materials (especially glass). In this glass system, the TKWN1 glass, doped only with Ho_2_O_3_, exhibited the reverse behavior, with a reduced density and molar volume. Previous reports [13,14] describe this unusual behavior for several glass systems containing rare-earth elements. It is well known that changes in molecular weight and density have an impact on the extent to which the molar volume changes. In comparison to the TKWN2 and TKWN3 glasses, the rate of change in the molar volume of the TKWN1 glass was lower. Hence, the network became more closed and tightly packed. This behavior could have been due to the addition of Er_2_O_3_ and Tm_2_O_3_ to the TKWN2 and TKWN3 glasses, respectively.

### 3.2. Thermal Properties

**Figure 2:**
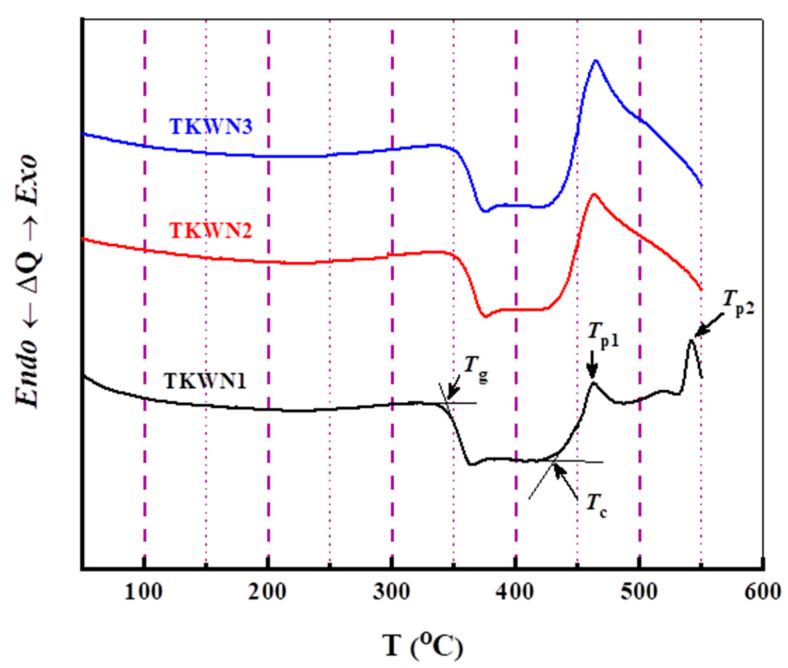
DSC curves of TKWN glasses at heating rate of 10 °C/min.

The DSC curves of the TKWN glasses doped with rare-earth oxides and tempered at a rate of 10 °C/min are illustrated in Figure 2. This glassy material’s thermal stability was confirmed by the DSC traces that were obtained for the samples. Each DSC scan exhibited a small endothermic peak corresponding to the glass transition temperature (*T*_g_), followed by exothermic peaks, with two peaks (*T*_p1_ and *T*_p2_) for the TKWN1 sample and one peak (*T*_p1_) for the TKWN2 and TKWN3 samples, which corresponded to the crystallization temperature. The glass transition temperature (*T*_g_), onset crystallization temperature (*T*_c_), and peak crystallization temperatures (*T*_p1_ and *T*_p2_) were measured and recorded, as shown in Table 2. The *T*_g_ provides information about the strength of the bonds and connectivity in the glass network, i.e., the *T*_g_ increases with the increasing connectivity and bond strength in the glass [15]. The values of *T*_g_ for the present glass system were close to those of TeO_2_-based glasses [16,17], which show high *T*_g_ values. The increase in the *T*_g_ values can result from the combined effect of incorporating both Nb_2_O_5_ and WO_3_ [18,19,20,21]. From Table 2, it is clear that the doping of the TKWN glasses with Er_2_O_3_ and Tm_2_O_3_ had a strong influence on the *T*_g_ as well as the onset and peak crystallization temperatures (*T*_c_ and *T*_p_), which shifted to significantly higher temperatures (as shown in Figure 2).

We found that adding Er_2_O_3_ and Tm_2_O_3_ to the TKWN glass doped with Ho_2_O_3_ improved the value of *T*_g_. This might have been because the bonds were very strong. Thus, the as-prepared glasses were more rigid and had better glass-forming capabilities after adding rare-earth oxides [14]. A further explanation for the reported increase in the *T*_g_ with increasing rare-earth oxide concentrations might be the OPD, as shown in Table 1. The OPD is a measure of the closeness of the oxide network’s packing. As the concentration of rare-earth oxides increases, it is evident that the OPD increases as well. This suggests that as the amount of rare-earth oxides grows, the structure becomes more compact. The presence of rare-earth oxides in the glass system suggests the production of a more compact macromolecular chain, which in return increases the *T*_g_, since a closer macromolecular structure requires greater internal energy for chain mobility, which is necessary for the glass transition [22].

An estimate for the thermal stability of glass has been calculated utilizing the thermal stability factor ∆*T* = (*T*_c_ − *T*_g_). To achieve the required large working range, e.g., during the fabrication process, it is favorable to have ∆*T* values that are as large as possible [23,24,25,26]. Hruby’s equation, namely *H* = ∆*T*/*T*_g_, and the glass compositional dependencies of Hruby’s coefficient were estimated by Sestak [27,28]. Table 2 displays Hruby’s coefficient (*H*) and the thermal stability factor (*∆T*); these are important in evaluating the glass devitrification process [29,30]. It was observed that the thermal stability of the studied glasses decreased with increasing rare-earth oxide concentrations. The parameter *K*_SP_, which is related to the stability of glass against crystallization, can be calculated using the following relationship [31]:

(2) $$K_{SP}=\frac{\left(T_{p}-T_{c}\right)\left(T_{p}-T_{g}\right)}{T_{g}}$$

where *T*_g_ is the glass transition temperature, *T*_c_ is the onset crystallization temperature, and *T*_p_ is the peak crystallization temperature.

Table 2 shows the KSP values for the as-prepared glasses, which lay within the range of those of tellurite-based glasses, which include alkaline and heavy metal ions, as reported in Refs. [16,31,32].

### 3.3. Optical Properties

**Figure 3:**
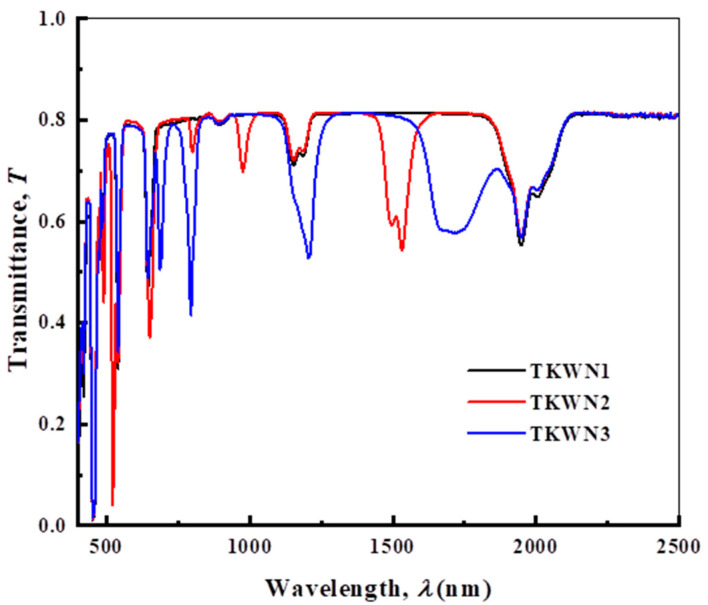
Optical transmission spectra of TKWN glasses.

**Figure 4:**
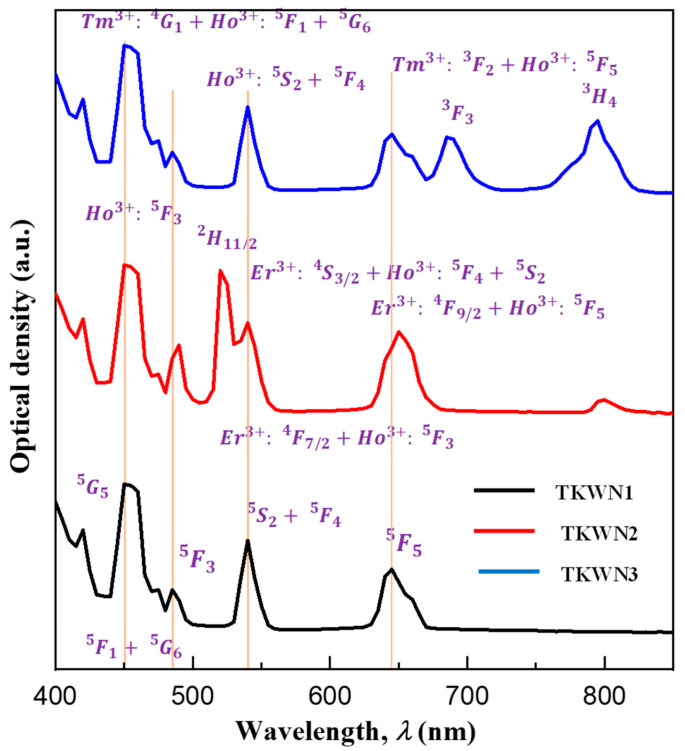
Room-temperature optical absorption spectra of TKWN glasses near UV–visible range.

**Figure 5:**
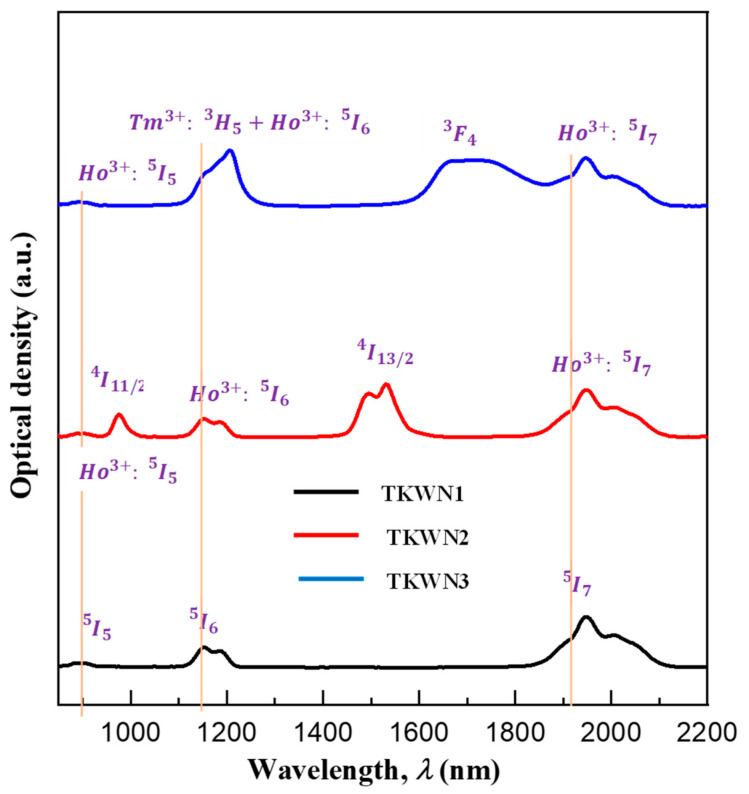
Room-temperature optical absorption spectra of TKWN glasses near infrared range.

The optical transmission spectra of the TKWN glasses doped with rare-earth oxides are illustrated in Figure 3. From this figure, we can see several peaks in the spectrum, which are due to the presence of rare-earth ions (Ho^3+^, Er^3+^, and Tm^3+^) in the glasses. Figure 4 and Figure 5 show the optical absorption spectra of the as-prepared glasses. Numerous peaks are due to the presence of rare-earth ions in the glass. The absorption coefficient (*α*) for the as-prepared glasses was calculated using the following equation [33]:

(3) $$\alpha =\frac{1}{d}\ln\left(\frac{I_{0}}{I_{t}}\right)=2.303\frac{A}{d}$$

where *I*_0_, *I_t_*, *A*, and *d* are the incident intensity, transmitted intensity, absorbance, and thickness of the film, respectively.

When designing devices that include glass, the material’s refractive index is an essential parameter that must be considered. The following equation expresses the relationship between the reflectance (*R*) and extinction coefficient (*k*) using the value of the real component of the complex refractive index (*n*), according to Fresnel’s theory of light reflectivity:

(4) $$R=\frac{{\left(n-1\right)}^{2}+k^{2}}{{\left(n+1\right)}^{2}+k^{2}}$$

The value of *k* can be calculated according to the following equation [34]:

(5) $$k=\frac{\alpha \lambda }{4\pi }$$

where *λ* is the wavelength in micrometers.

**Figure 6:**
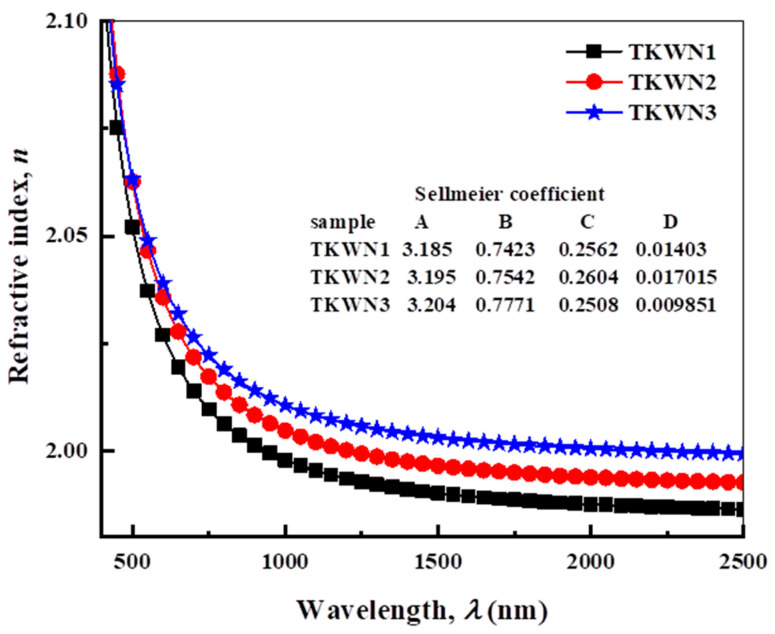
Dependence of refractive index (n) on wavelength (λ) for TKWN glasses.

**Figure 7:**
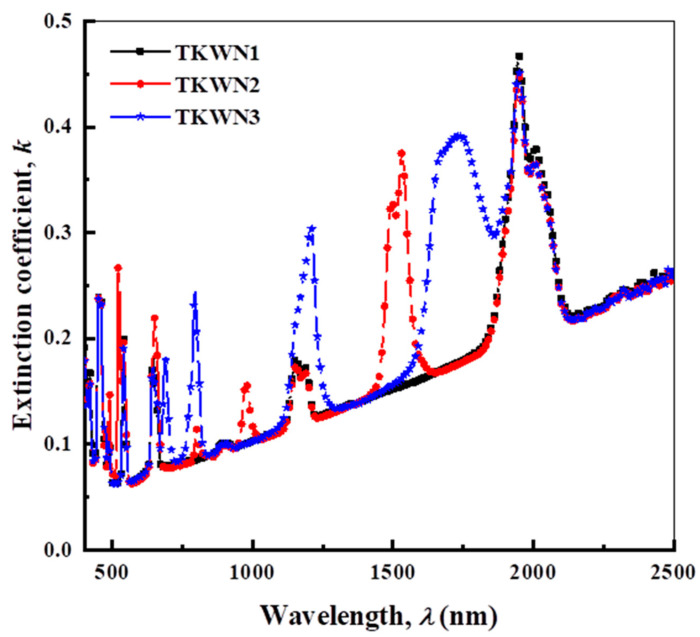
Dependence of extinction coefficient (k) on wavelength (λ) for TKWN glasses.

The calculated *n* and *k* values of the TKWN glasses doped with rare-earth oxides are given in Figure 6 and Figure 7, respectively. As shown in Figure 6, the refractive index (*n*) decreased when increasing the wavelength of the incident photon.

The density, the electronic polarizability of the oxide ion, the coordination number, and the polarizability of the initial neighbor ions coordinated with it (anions) are among the several important variables that impact the refractive index [21]. The Er_2_O_3_ or Tm_2_O_3_ added to the TKWN glass doped with Ho_2_O_3_ caused a minor increase in the *n* values. In addition, we found that the density, *ρ*, and *n* had a linear relationship. See Figure 6 for the highest values of *n* for the TKWN glass samples doped with both Ho_2_O_3_ and Tm_2_O_3_.

The Sellmeier dispersion formula is one of the most well-known fitted dispersion equations that describes the index variation, *n*, vs. the wavelength, λ. The five coefficients included in this formula allow it to fit the data perfectly throughout a wide spectrum range, in agreement with the observations. One means to describe the propagation characteristics of waveguides constructed from the materials under study is to employ the dispersion data in the form of fitting Sellmeier coefficients. The following is the Sellmeier dispersion formula in the spectra of the absorption bands; when (*hν*) is smaller than (*E*_opt_), the photon band gap energy is as follows [35,36]:

(6) $$n^{2}\left(\lambda \right)=A+B/\left(1-\frac{C}{\lambda^{2}}\right)+D/\left(1-\frac{E}{\lambda^{2}}\right)$$

where *λ* is the wavelength in micrometers. In this case, the glass materials’ dispersion characteristics are *A*, *B*, *C*, *D*, and *E*. The first and second terms relate to the refractive index contributions of larger and lower energy gaps from electronic absorption. The last term indicates how the lattice absorption causes the refractive index to decrease [35,36]. Equation (6) was used to fit the experimental data, yielding the Sellmeier coefficients shown in Table 3. Figure 6 shows the refractive index behavior for the as-prepared glasses with the wavelength calculated using Sellmeier’s equations [35].

**Figure 8:**
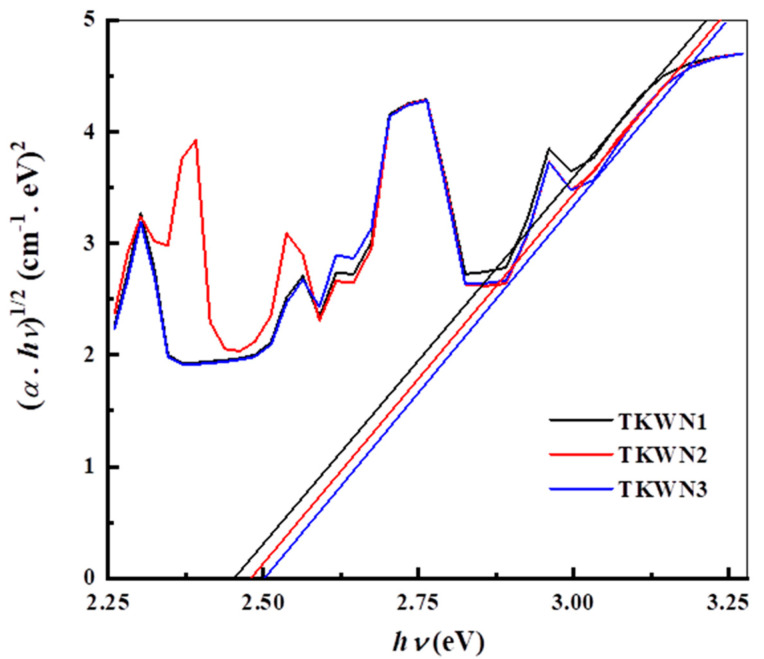
Plot of (αhν)^1/2^ against hν for TKWN glasses.

Furthermore, $E_{g}=1.24/$λ is the average absorption band gap, *E_g_* (measured in electron volts), and it may be used to determine the lattice absorption frequency or *E_g_* [35]. For more information about optically induced transitions and optical band gaps in materials, it is helpful to investigate their optical absorption edges. The basic idea behind this processing is the absorption of photons whose energies are higher than the energy of the band gap. At the basic absorption edge, electromagnetic waves interact with electrons in the valence band to cause two types of optical transitions: direct and indirect. The Tauc relation [37] provides the relationship between α and the photon energy of the incoming radiation, *hν*.

(7) $$\alpha \;h\nu =b{\left(h\nu -E_{opt}\right)}^{s}$$

where *ν* is the frequency and *h* is the Planck constant. While *b* remains constant, the value of *s* varies according to the interband transition process. The parameter *s* takes the value of ½ in the case of the direct allowed transition, while it is equal to 2 in the case of the indirect allowed transition. Equation (7), which is associated with indirect permitted transitions in most types of glass, shows a straight line for s = 2. The Tauc plot of (*αhν*)^1/2^ against (*hν*) for the as-prepared glasses is shown in Figure 8. To determine the *E*_opt_ of these glasses, we extrapolated their linear domains at the absorption edge to intersect the hν axis at (*αhν*)^1/2^ = 0. Table 3 lists the *E*_opt_ values. Adding Er_2_O_3_ or Tm_2_O_3_ to the TKWN glass doped with Ho_2_O_3_ slightly increased the *E*_opt_ value; it was greatest in the TKWN glass sample that was co-doped with Ho_2_O_3_ and Tm_2_O_3_. As mentioned previously regarding the thermal properties and density of the proposed glass material, the strong bonds are the expected cause of the increase in the *E*_opt_. values. The results for the present glass material show that when the amount of rare-earth oxides is increased, the structure becomes more closely packed, leading to an increase in the *T_g_*, ρ, and OPD. Hence, increasing the number of rare-earth oxides in the glass system is suggested to form a more rigid macromolecular chain, which decreases the amount of non-bridging oxygen (NBO) and increases the *E*_opt_.

**Figure 9:**
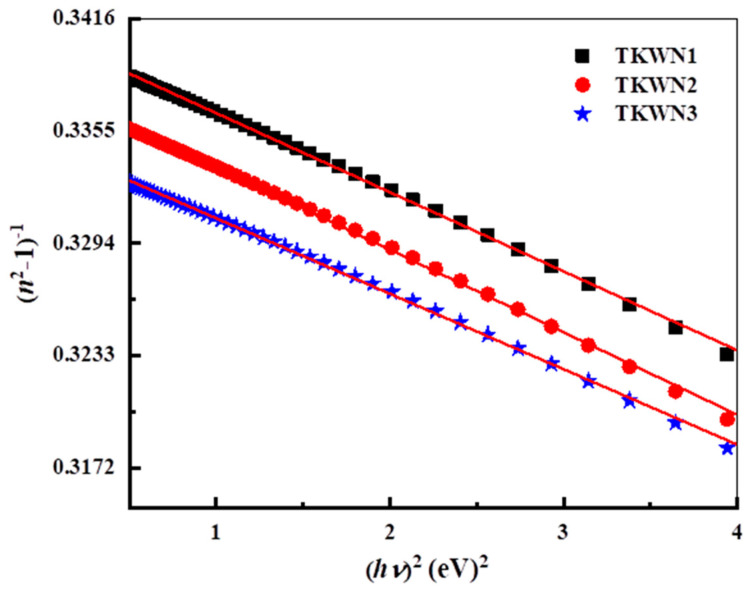
Plot of (n^2^ − 1)^−1^ as a function of (hʋ)^2^ for TKWN glasses.

**Figure 10:**
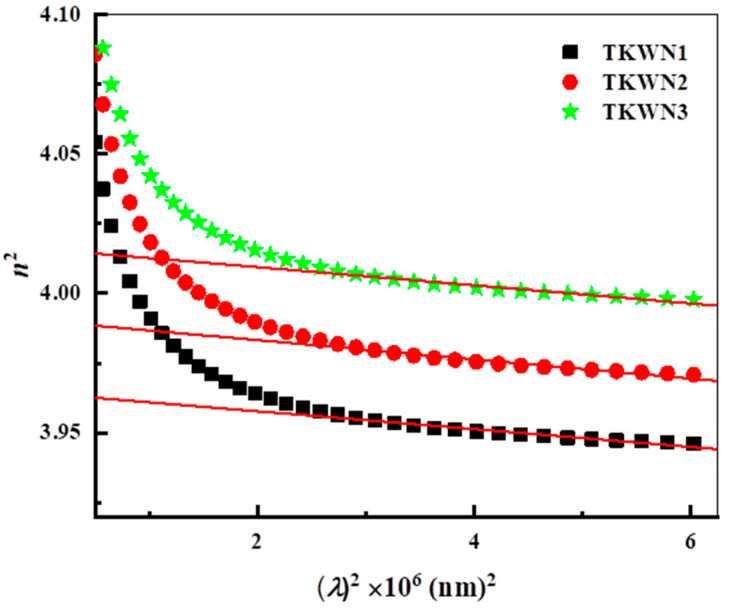
Variation in *n*^2^ as a function of *λ*^2^ for TKWN glasses.

By applying the Wemple–DiDomenico (WDD) relationship to the model of a single oscillator, we may describe the dispersion of n [38].

(8) $$n^{2}=1+\left(\frac{E_{d}E_{o}}{E_{o}^{2}-{\left(h\nu \right)}^{2}}\right)$$

where *E*_d_ is the dispersion energy, which represents the average strength of the interband optical transitions, and *E*_o_ is the energy of the effective dispersion oscillator or the average energy gap. Figure 9 shows the variation in (*n*^2^ − 1)^−1^ versus (*hν*)^2^ for the studied glasses. The values of *E*_d_ and *E*_o_ can be directly determined from the slope (*E*_o_*E*_d_)^−1^ and the intercept on the vertical axis (*E*_d_/*E*_o_). The static refractive index (*n*_0_) of the as-prepared glasses is calculated via the extrapolation of the Wemple–DiDomenico dispersion relation, Equation (8), when *hν*→0, and this gives the following expression:

(9) $$n_{0}=\sqrt{1+E_{d}/E_{o}}\;$$

where *n*_o_ is the static refractive index. The deduced values of *E*_o_, *E*_d_, and *n*_o_ are listed in Table 4. It is observed that the value of *n*_o_ for the as-prepared glasses increases with an increase in density. Figure 10 shows that *n*^2^ is strongly dependent on *λ*^2^ according to Equation (6).

A suitable method for the investigation of the impact of ionic packing on the refractive index (*n*) of glass is to calculate its molar refractivity (*R*_m_), which is defined as the total polarizability of a mole of a material and derived from the following formula [33]:

(10) $$R_{m}=\left(\frac{n_{o}^{2}-1}{n_{o}^{2}+2}\right)V_{m}$$

where *V_m_* is the molar volume.

According to the following Clasius–Mosotti relationship, the molar electronic polarizability of a material is proportional to its molar refractivity, which, in effect, corresponds to the glass’s structure [33].

(11) $$\alpha_{m}=\left(\frac{3}{4\pi N_{A}}\right)R_{m}$$

where *N*_A_ is Avogadro’s number. The values of *R*_m_ and *α*_m_ are listed in Table 3. These values increase with the increase in the rare-earth content. The metallization criterion (*M*) gives information about the metallic or non-metallic nature of the glass, and it is calculated through the following relationship [31]:

(12) $$M=1-\frac{R_{m}}{V_{m}}$$

If *M* > 0, the materials demonstrate an insulating nature, but if *M* < 0, the materials exhibit a metallic nature. The results of the metallization criterion (*M*) are listed in Table 3 and are within the range of 0.501–0.506. Therefore, the as-prepared glasses demonstrate an insulating nature [16,31].

### 3.4. Absorption Spectra, Judd–Ofelt Analysis, and Radiative Properties

Extrinsic absorption, which is associated with internal electronic transitions, particularly in the 4f shells of rare-earth ions, and intrinsic absorption, which occurs at short and long wavelengths, are the two processes that lead to the development of the optical spectra of single-rare-earth-doped or co-doped glasses [39,40].

The room-temperature UV–Vis–NIR absorption spectra recorded in the range of 400–2500 nm for the Ho^3+^-single-doped, Ho^3+^/Er^3+^-co-doped, and Ho^3+^/Tm^3+^-co-doped tellurite glasses (TKWN1, TKWN2, and TKWN3, respectively) and the absorption peaks related to electronic transitions from the ground states of Ho^3+^, Er^3+^, and Tm^3+^ to their corresponding excited levels are presented in Figure 4 and Figure 5**,** respectively. The eight absorption peaks from the ground state Ho^3+^ are responsible for the bands at the wavelengths of 420, 450, 485, 540, 645, 890, 1155, and 1950 nm for Ho^3+^: ^5^*I*_8_ regarding the degree of intensity; correspondingly, the resulting values are ^5^*G*_5_, ^5^*F*_1_+^5^*G*_6_, ^5^*F*_3_, ^5^*S*_2_+^5^*F*_4_, ^5^*F*_5_, ^5^*I*_5_, ^5^*I*_6_, and ^5^*I*_7_. The seven absorption peaks from the ground state Er^3+^ are Er^3+^: ^4^*I*_15/2_ to the excited levels ^4^F_7/2_, ^2^*H*_11/2_, ^4^*S*_3/2_, ^4^*F*_9/2_, ^4^*I*_9/2_, ^4^*I*_11/2_, and ^4^*I*_13/2_, respectively. The bands with peaks at the wavelengths of 450, 645, 685, 795, and 1710 nm are derived from the five absorption peaks that start the transition of the ground state Tm^3+^, namely Tm^3+^: ^3^*H*_6_ to the excited levels ^1^G_4_, ^3^F_2_, ^3^F_3_, ^3^H_3_, ^3^H_5_, and ^3^F_4_, respectively. These are identical to the absorption peaks seen in other glasses [41,42,43].

Figure 4 further confirms that the presence of Er^3+^ or Tm^3+^ ions in the matrix causes absorption bands to form due to the energy-level quantum structures in these ions [44]. In photoluminescence measurements, the 808 nm commercial laser diode (LD) can be utilized as a pumping source because although Ho^3+^ ions do not exhibit any clear absorption peaks around 808 nm or 980 nm, the ^3^*H*_4_ ground state of Tm^3+^ and the ^4^I_9/2_ ground state of Er^3+^ both exhibit an absorption peak at around 800 nm. The locations of the absorption peaks exhibit no apparent variations when compared to the Ho^3+^-single-doped sample (TKWN1). In addition, the two Ho^3+^/Er^3+^-co-doped tellurite glasses (TKWN2 and TKWN3, respectively) match the tellurite glass samples in terms of the forms and peak locations of each transition [45,46,47]. In order to investigate the potential spectroscopic parameters of the single-doped sample (TKWN1), the Judd–Ofelt (JO) theory is adopted without taking into account the energy transfer and multiphonon de-excitation probabilities. Detailed applications of the JO model have been described in other papers [48,49]. We compute the experimental electric dipole line strength $\left(S_{ed}^{mes}\right)$ of the Ho^3+^-single-doped glass sample (TKWN1) using the absorbance spectra (Figure 4 and Figure 5). Unlike the glasses doped with other rare-earth ions, such Er^3+^ and Tm^3+^, the magnetic dipole transitions in the Ho^3+^-ion-doped glass are sufficient to be undetectable [50]. Thus, the magnetic dipole transitions should be taken into account when we calculate the experimental electric dipole line strength ${(S}_{ed}^{mes})$. The calculated values are given in Table 5, along with the values of the magnetic dipole line strength $S_{md}^{\;}$, the three phenomenological intensity parameters $\Omega_{t}\;(t=2,\;4,\;6)$, and the calculated electric dipole line strength ${(S}_{ed}^{cal})$. As shown in Table 5, good agreement is found between the calculated and the experimental values, and the lower value of the root mean square deviation between the experimental and the calculated line strengths of the transitions $\left(\delta_{rms}=0.5659\times 10^{-20}\;{cm}^{2}\right)$ indicates the validity of JO theory for the prediction of the spectral intensity of Ho^3+^.

Hypersensitive transitions (HSTs) are transitions associated with all absorptions, e.g., Ho^3+^: *^5^I_8_*→*^5^G_5_* transitions. These transitions are very sensitive to the surrounding local environment of the doped ions and follow the selection criteria ΔL≤2, ΔJ≤2, and ΔS = 0 [51,52,53].

It is well known that parameter $\Omega_{2}$ represents HSTs and is dependent on the short-range effects of rare-earth ions (the covalency and asymmetry in the community). The ion site is more centro-symmetric and its chemical interaction with the ligand is more ionic when the value of $\Omega_{2}$ is smaller, both of which contribute to the covalent nature [54]. The massive (bulk) characteristics of the host glass, such as its basicity and strength, are associated with the constants $\Omega_{4}$ and $\Omega_{6}$. In addition, the vibrational levels related to the essential rare-earth ions confined to the ligand atoms have a significant impact on them [55,56,57]. The host glass’s basicity and hardness increase with increasing values of $\Omega_{4}$ and $\Omega_{6}$. The three Ho^3+^ intensity parameters follow the trend $\Omega_{4}$ > $\Omega_{2}$ > $\Omega_{6}$, according to an analysis of the data in Table 5.

The spectroscopic quality factor $\left(\chi =\Omega_{4}/\Omega_{6}\right)$ is an important characteristic in predicting the luminescence efficiency [58]. In the sample TKWN1, the value calculated is 1.93, which is higher than those of previously reported Ho^3+^-single-doped samples [59,60].

To predict the emission performance in the studied glass (TKWN1), the radiative properties, such as the radiative transition probabilities ($A_{rad}\left(J\to J\prime \right)=A_{ed}+A_{md})$, branching ratios $\left(\beta_{rad}\left(JJ\prime \right)\right)$, and radiative lifetimes $\left(\tau_{rad}\right)$, for the $\left(J\to J\prime \right)$ transitions for spontaneous emission are calculated through JO intensity parameters. All data computed are tabulated in Table 6. Compared to fluorophosphate ($90.42\;s^{-1})$ [61], tellurite ($165.8\;s^{-1}$) [60], and germanate glass ($69.2\;s^{-1}$) [62], the TKWN1 sample examined in this study has $A_{rad}$ in which the Ho^3+^→^5^*I*_7_→^5^*I*_8_ transition was high and equal to $38.7\;s^{-1}$. This is determined by the higher refractive index (n=1.98) of the tellurite glass, because the larger refractive index of the host glass, the higher the radiative transition probability, which provides a better likelihood of achieving laser action [63]. Thus, the TKWN1 is possibly a suitable material that might be able to achieve 2 μm fluorescence via the Ho^3+^: ^5^*I*_7_ → ^5^*I*_8_ transition.

**Figure 11:**
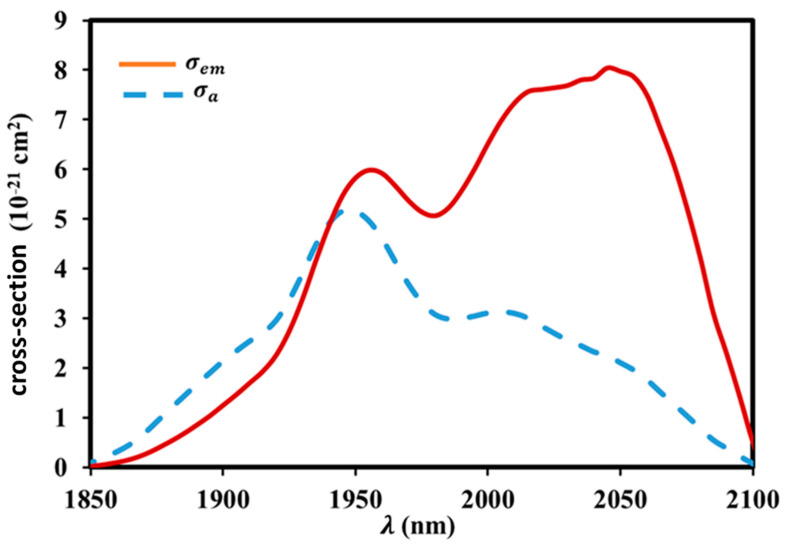
Calculated absorption and emission cross-sections for I75→I85 transition of Ho^3+^-ion-doped tellurite glass (TKWN1).

As a key parameter influencing the potential laser performance, the absorption ($\sigma_{a}$) and emission $\left(\sigma_{e}\right)$ cross-sections need to be calculated. The absorption cross-sections corresponding to the ^5^*I*_7_ →^5^*I*_8_ transition of Ho^3+^ are first determined from the measured absorption spectra using the Beer–Lambert equation [64,65], while the emission cross-section for the ^5^*I*_7_ →^5^*I*_8_ transition of Ho^3+^ is evaluated from the obtained absorption cross-sections based on McCumber’s theory [66]. The calculated absorption and emission cross-sections of Ho^3+^ in the range of 1850–2100 nm are displayed in Figure 11. The absorption and emission maxima are located at 1950 nm and 2045 nm, according to Figure 11.

Therefore, the peak value of the stimulated emission cross-section is approximately $8\times 10^{-21}\;{cm}^{2}$. It is smaller than the estimated values for bismuth glass ($10.09\times 10^{-21}\;{cm}^{2}$) [67] and tellurite glass ($10\times 10^{-21}\;{cm}^{2}$) [68], but it is far larger than that of silicate ($3.54\times 10^{-21}\;{cm}^{2}$) [69], fluoride ($2.47\times 10^{-21}\;{cm}^{2}$) [70], and germanate glass ($3.13\times 10^{-21}\;{cm}^{2}$) [60].

There is a direct correlation between the refractive index of the host glass and the emission cross-section; a higher refractive index increases the possibility of spontaneous radiative transitions. The higher spontaneous radiative transition probability and high refractive index are the primary factors that cause the Ho^3+^ in the produced glass to have an extended emission cross-section.

Additionally, the $FWHM\times \sigma_{e}$ value is a significant indicator that is often used to define the gain characteristics; the TKWN1 sample corresponds with larger gain properties and a wider gain bandwidth with a higher gain quality value. This study computed that the emission cross-section ($\sigma_{e}$) and *FWHM* are $8\times 10^{-21}\;{cm}^{2}$ and 150 nm, respectively. Furthermore, as Table 7 illustrates, the $FWHM\times \sigma_{e}$ is $1200\times 10^{-28}\;{cm}^{3}$ greater than that of a variety of glasses [71]. The TKWN1 sample may have promise as a laser material, according to these findings.

**Figure 12:**
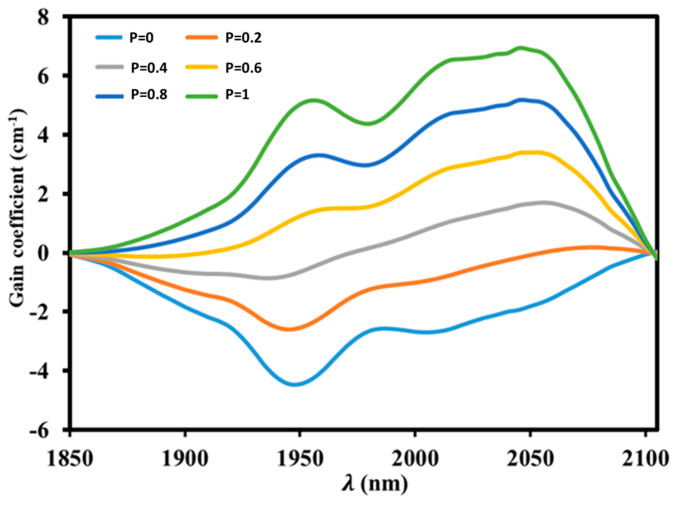
Gain coefficient for I75→I85 transition of Ho^3+^-single-doped tellurite glass (TKWN1).

The wavelength-dependent gain cross-section, which establishes the gain spectrum shape and amplification performance, may be computed using the formula given in a previous article [72] based on the absorption and emission cross-sections of Ho^3+^. Figure 12 shows the computed gain cross-section spectra of the Ho^3+^: I75→I85 radiative transition in the Ho^3+^-single-doped tellurite glass, with a population inversion value P ranging from 0 to 1 and with an interval of 0.2. It is clear from this spectrum that as the inversion population develops, the gain region moves to longer wavelengths and the gain cross-section improves.

When compared to germanate glass ($0.47\;{cm}^{-1}$), tellurite glass ($1.2\;{cm}^{-1})$, and fluorophosphate glass ($0.66\;{cm}^{-1}$), the highest gain cross-section at 2045 nm is $6.94\;{cm}^{-1}$ [73,74,75]. A positive gain coefficient indicates a low pump threshold for laser operation when P is 0.6 and λ is greater than 1910 nm [76]. A quasi-three-level system is characterized by an extended positive gain band that increases with P [77].

## 4. Conclusions

Systematic investigations have been performed to understand the effects of Ho_2_O_3_, Er_2_O_3_, and Tm_2_O_3_ doping on the thermal and optical properties of TeO_2_-K_2_TeO_3_-WO_3_-Nb_2_O_5_ glass. The DSC spectra confirmed the amorphous nature of the as-prepared glasses. Moreover, the DSC spectra showed that the glass transition temperature increased with the incorporation of Er_2_O_3_ and Tm_2_O_3_ into the TeO_2_-K_2_TeO_3_-WO_3_-Nb_2_O_5_ glass doped with Ho_2_O_3_. The thermal stability was investigated through the thermal stability factor ∆*T*, Hruby’s coefficient *H*, and parameter *K*_SP_. The data obtained on the transmittance *T* and absorbance *A* for the as-prepared glasses were used to calculate the absorption index *α*, the refractive index *n*, the extinction coefficient k, and the optical band gap *E*_opt_. The optical band gap *E*_opt_ and refractive index *n* slightly increased with the incorporation of Er_2_O_3_ and Tm_2_O_3_ into the TeO_2_-K_2_TeO_3_-WO_3_-Nb_2_O_5_ glass doped with Ho_2_O_3_. Furthermore, the TeO_2_-K_2_TeO_3_-WO_3_-Nb_2_O_5_ glass sample doped with both Ho_2_O_3_ and Tm_2_O_3_ displayed the highest values for these parameters. The UV–Vis–NIR absorption and emission spectra of the Ho^3+^-single-doped, Ho^3+^/Er^3+^-co-doped, and Ho^3+^/Tm^3+^-co-doped tellurite glasses (TKWN1, TKWN2, and TKWN3, respectively) were examined at room temperature.

The Judd–Ofelt theory was used to determine the full spectroscopic characteristics, radiative lifetimes, branching ratios, and radiative transition probabilities of the TKWN1 sample. Furthermore, McCumber’s theory was used to compute the absorption and emission cross-sections of Ho^3+^: ^5^*I*_7_ →^5^*I*_8_. There was a maximum emission cross-section of $8\times 10^{-21}\;{cm}^{2}$. Additionally, the gain coefficient of Ho^3+^, ^5^*I*_7_ →^5^*I*_8_, the quality factor ($\sigma_{e}^{\;}\times FWHM)$, and the *FWHM* were studied. The $FWHM\times \sigma_{e}$ value was $1200\times 10^{-28}\;{cm}^{3}$, indicating that alternative glasses have greater gain characteristics. Hence, the TKWN1 sample has potential to be utilized as a host material for 2 μm mid-infrared solid-state lasers.

## Figures and Tables

**Table 1:** Composition, density *ρ*, molar volume V_m_, and oxygen packing density (OPD) of TKWN glasses.

Sample	Composition (mol%)	ρ (g/cm^3^)	V_m_ (cm^3^/mol)	OPD (mol/L)
TKWN1	60TeO_2_-20K_2_TeO_3_-10WO_3_-10Nb_2_O_5_-30,000 ppmHo_2_O_3_	4.9125	41.07	65.51
TKWN2	60TeO_2_-20K_2_TeO_3_-10WO_3_-10Nb_2_O_5_-30,000 ppmHo_2_O_3_-30,000 ppm Er_2_O_3_	4.9597	41.80	66.51
TKWN3	60TeO_2_-20K_2_TeO_3_-10WO_3_-10Nb_2_O_5_-30,000 ppmHo_2_O_3_-30,000 ppm Tm_2_O_3_	4.9641	41.78	66.53

**Table 2:** Values of *T*_g_, *T*_c_, *T*_p_, ∆*T*, *H*, and *KSP* for TKWN glasses.

Sample Code	*T*_g_ °C	*T*_c_ °C	*T*_p1_ °C	*T*_p2_ °C	∆*T* °C	*H*	*K*_SP_ °C
**TKWN1**	344	431	462	542	87	0.253	10.63
**TKWN2**	355	438	463	-	83	0.234	7.61
**TKWN3**	356	439	464	-	83	0.233	7.58

**Table 3:** Values of *A*, *B*, *C*, *D*, *E*_opt_, *R*_m_, α_m_, and *M* for TKWN glasses.

Sellmeier Coefficients					*E*_opt_ (eV)	*R*_m_ Mol^−1^	*α*_m_ Å^−3^	*M*
Sample Code	*A*	*B*	*C*	*D*				
TKWN1	3.185	0.7423	0.2562	0.01403	2.453	20.30	8.05	0.506
TKWN2	3.195	0.7542	0.2604	0.017015	2.481	20.75	8.23	0.504
TKWN3	3.204	0.7771	0.2508	0.009851	2.501	20.84	8.26	0.501

**Table 4:** Optical parameters of TKWN glasses.

	Dispersion Parameters		
Sample Code	*E*_o_ (eV)	*E*_d_ (eV)	*n*_o_
TKWN1	8.60	25.25	1.983
TKWN2	8.68	25.69	1.989
TKWN3	9.03	26.98	1.996

**Table 5:** Values of average wavelengths, refractive indices, integrated absorption coefficients, and electric and magnetic dipole line strengths for Ho^3+^-single-doped tellurite glass (TKWN1).

Transition from I85 to	$\bar{\lambda }\;(nm)$	n	$\int OD\left(\lambda \right)d\lambda \;(nm)$	$S_{ed}^{cal}\;(pm^{2})$	$S_{ed}^{mes}\;(pm^{2})$	$S_{md}\;(pm^{2})$
^5^ *I*_7_	1950	1.9884	25.9613	1.9518	1.2918	0.9493
^5^ *I*_6_	1155	1.9959	5.0289	0.8340	1.0327	0
^5^ *I*_5_	895	2.0041	0.52004	0.1202	0.1370	0
^5^ *F*_5_	645	2.0226	6.0251	1.4788	2.1687	0
^5^*S*_2_+^5^*F*_4_	540	2.0420	4.65323	1.4749	1.9679	0
^5^ *F*_3_	485	2.0609	0.92585	0.3694	0.4291	0
^5^F_1_ + ^5^G_6_	450	2.0790	9.9929	4.9075	4.9172	0
^5^ *G*_5_	420	2.1003	0.89175	1.0969	0.4619	0
$\Omega_{2}=1.993\times 10^{-20}\;{cm}^{2}$			$\Omega_{4}=2.055\times 10^{-20}\;{cm}^{2}$		$\Omega_{6}=1.066\times 10^{-20}\;{cm}^{2}$	
$\delta_{rms}=0.5659\times 10^{-20}{cm}^{2}$						

**Table 6:** Calculated radiative parameters of different states of Ho^3+^-single-doped tellurite glass (TKWN1).

Transition	$\bar{\nu }\;({cm}^{-1})$	n	$S_{ed}\;(pm^{2})$	$S_{md}\;(pm^{2})$	$A_{ed}\;(s^{-1})$	$A_{md}\;(s^{-1})$	β (%)	$\tau_{r}\;(ms)$
^ 5 ^ *I*_7_ → ^5^ *I*_8_	5102	1.9884	1.9505	0.8670	195	43.6554	100.0000	4.1983
^ 5 ^ *I_6_* → ^5^ *I*_8_	8658	1.9959	0.8305	0	475	0	86.1550	1.8124
^ 5 ^ *I*_6_ → ^5^ *I*_7_	3556	1.9874	1.3362	1.2450	52	24.4519	13.8450	
^ 5 ^ *I*_5_ → ^5^ *I*_8_	11148	2.0040	0.1201	0	177	0	40.9162	2.3152
^ 5 ^ *I* *_5_* → ^5^ *I*_7_	6046	1.9897	0.9896	0	225	0	52.0198	
^ 5 ^ *I*_5_ → ^5^ *I*_ 6 _	2612	1.9873	1.0679	1.2050	19	11.0797	7.0640	
^ 5 ^ *I*_4_ → ^5^ *I*_8_	13333	2.0124	0.0082	0	26	0	7.6306	2.9607
^ 5 ^ *I*_4_ → ^5^ *I*_7_	8231	1.9947	0.1720	0	22	0	36.0841	
^ 5 ^ *I*_4_ → ^5^ *I*_ 6 _	4675	1.9880	1.3163	0	168	0	49.8021	
^ 5 ^ *I*_4_ → ^5^ *I*_ 5 _	2185	1.9872	1.2959	0.7640	17	5.0263	6.4831	
^ 5 ^ *F*_5_ → ^5^ *I*_8_	15504	2.0226	1.5178	0	6272	0	78.3197	0.1249
^ 5 ^ *F*_5_ → ^5^ *I*_7_	10402	2.0014	1.2166	0	1446	0	18.0502	
^ 5 ^ *F*_5_ → ^5^ *I*_ 6 _	6846	1.9913	0.8152	0	270	0	3.3668	
^ 5 ^ *F*_5_ → ^5^ *I*_ 5 _	4356	1.9877	0.2474	0	21	0	0.2610	
^ 5 ^ *F*_5_ → ^5^ *I*_4_	2171	1.9872	0.0174	0	0	0	0.0023	
^ 5 ^ *S*_2_ → ^5^ *I*_8_	18882	2.0449	0.2202	0	3808	0	55.6069	0.1460
^ 5 ^ *S*_2_ → ^5^ *I*_ 7 _	13080	2.0114	0.4431	0	2357	0	34.4197	
^ 5 ^ *S*_2_ → ^5^ *I*_ 6 _	9524	1.9986	0.2033	0	405	0	5.9170	
^ 5 ^ *S*_2_ → ^5^ *I*_ 5 _	7034	1.9917	0.1105	0	87	0	1.2745	
^ 5 ^ *S*_2_ → ^5^ *I*_4_	4848	1.9881	0.7365	0	189	0	2.7603	
^ 5 ^ *S*_2_ → ^5^ *F*_5_	2678	1.9873	0.0345	0	1	0	0.0217	
^ 5 ^ *F*_4_ → ^5^ *I*_8_	18868	2.0448	1.2702	0	12173	0	79.3540	0.0652
^ 5 ^ *F*_4_ → ^5^ *I*_ 7 _	13766	2.0143	0.4581	0	1589	0	10.3557	
^ 5 ^ *F*_4_ → ^5^ *I*_ 6 _	10210	2.0008	0.7410	0	1016	0	6.6243	
^ 5 ^ *F*_4_ → ^5^ *I*_ 5 _	7720	1.9933	0.7804	0	455	0	2.9631	
^ 5 ^ *F*_4_ → ^5^ *I*_4_	5535	1.9889	0.3239	0	69	0	0.4486	
^ 5 ^ *F*_4_ → ^5^ *F*_5_	3364	1.9873	0.6371	0.3620	30	8.6944	0.2541	
^ 5 ^ *F*_4_ → ^5^ *S*_2_	686	1.9872	0.0400	0	0	0	0.0001	0.0740
^ 5 ^ *F*_3_ → ^5^ *I*_8_	20619	2.0609	0.3649	0	6088	0	45.0747	
^ 5 ^ *F*_3_ → ^5^ *I*_ 7 _	15517	2.0226	0.7745	0	5043	0	37.3366	
^ 5 ^ *F*_3_ → ^5^ *I*_ 6 _	11961	2.0070	0.4212	0	1211	0	8.9682	
^ 5 ^ *F*_3_ → ^5^ *I*_ 5 _	9470	1.9984	0.4917	0	688	0	5.0934	
^ 5 ^ *F*_3_ → ^5^ *I*_4_	7285	1.9923	0.6292	0	395	0	2.9249	
^ 5 ^ *F*_4_ → ^5^ *F*_5_	5115	1.9884	0.3492	0	75	0	0.5567	
^ 5 ^ *F*_3_ → ^5^ *S*_2_	2437	1.9872	0.0153	0	0	0	0.0026	
^ 5 ^ *F*_3_ → ^5^ *F*_4_	1751	1.9872	0.3772	0.5800	3	2.5237	0.0427	

**Table 7:** Comparison of FWHM, $\sigma_{e}$, and $\sigma_{e}\times FWHM$ values for I75→I85 transition of TKWN1.

	FWHM (nm)	$\sigma_{e}\;(cm^{2})$	$\sigma_{e}\times FWHM\;(cm^{3})$	Reference
TKWN1	150	$8.0\times 10^{-21}$	$1200\times 10^{-28}$	Present work
Silicate	82	$5.5\times 10^{-21}$	$220\times 10^{-28}$	[71]
Phosphate	78	$6.4\times 10^{-21}$	$236.8\times 10^{-28}$	[71]
Germanate	84	$4.0\times 10^{-21}$	$336\times 10^{-21}$	[71]

## Data Availability

The original contributions presented in the study are included in the article, further inquiries can be directed to the corresponding author.
